# Supplementary material for: Ocean fronts as decadal thermostats modulating continental warming hiatus
Source: Nat Commun. 2023 Nov 27;14:7777. doi: 10.1038/s41467-023-43686-1 (PMC10682185; doi:10.1038/s41467-023-43686-1)
Supplement: Supplementary file 1 — Supplementary Information [file 41467_2023_43686_MOESM1_ESM.docx]

Supplementary Information for

Ocean fronts as decadal thermostats modulating continental warming hiatus

Mi-Kyung Sung^1^*, Soon-Il An^2,7^*, Jongsoo Shin^3^, Jae-Heung Park^4^, Young-Min Yang^5^, Hyo-Jeong Kim^6^ and Minhee Chang^1^

^1^Climate and Environmental Research Institute, Korea Institute of Science and Technology; Seoul, Republic of Korea

^2^Department of Atmospheric Sciences/Irreversible Climate Change

Research Center, Yonsei University; Seoul, Republic of Korea

^3^Woods Hole Oceanographic Institution; Woods Hole, Massachusetts, USA

^4^School of Earth and Environmental Sciences, Seoul National University; Seoul, Republic of Korea

^5^Key Laboratory of Meteorological Disaster, Ministry of Education (KLME)/Joint International Research Laboratory of Climate and Environment Change (ILCEC)/Collaborative Innovation Center on Forecast and Evaluation of Meteorological Disasters (CIC-FEMD), Nanjing University of Information Science and Technology; Nanjing, China

^6^Low-Carbon and Climate Impact Research Centre, School of Energy and Environment, City University of Hong Kong; Hong Kong SAR, China

^7^Division of Environmental Science and Engineering, Pohang University of Science and Technology, Pohang, Republic of Korea

*Corresponding author. Email: [sian@yonsei.ac.kr](mailto:sian@yonsei.ac.kr) (S.-I. A.); [mksung@kist.re.kr](mailto:mksung@kist.re.kr) (M.-K. S.)

The SI includes Supplementary Figures 1-7.


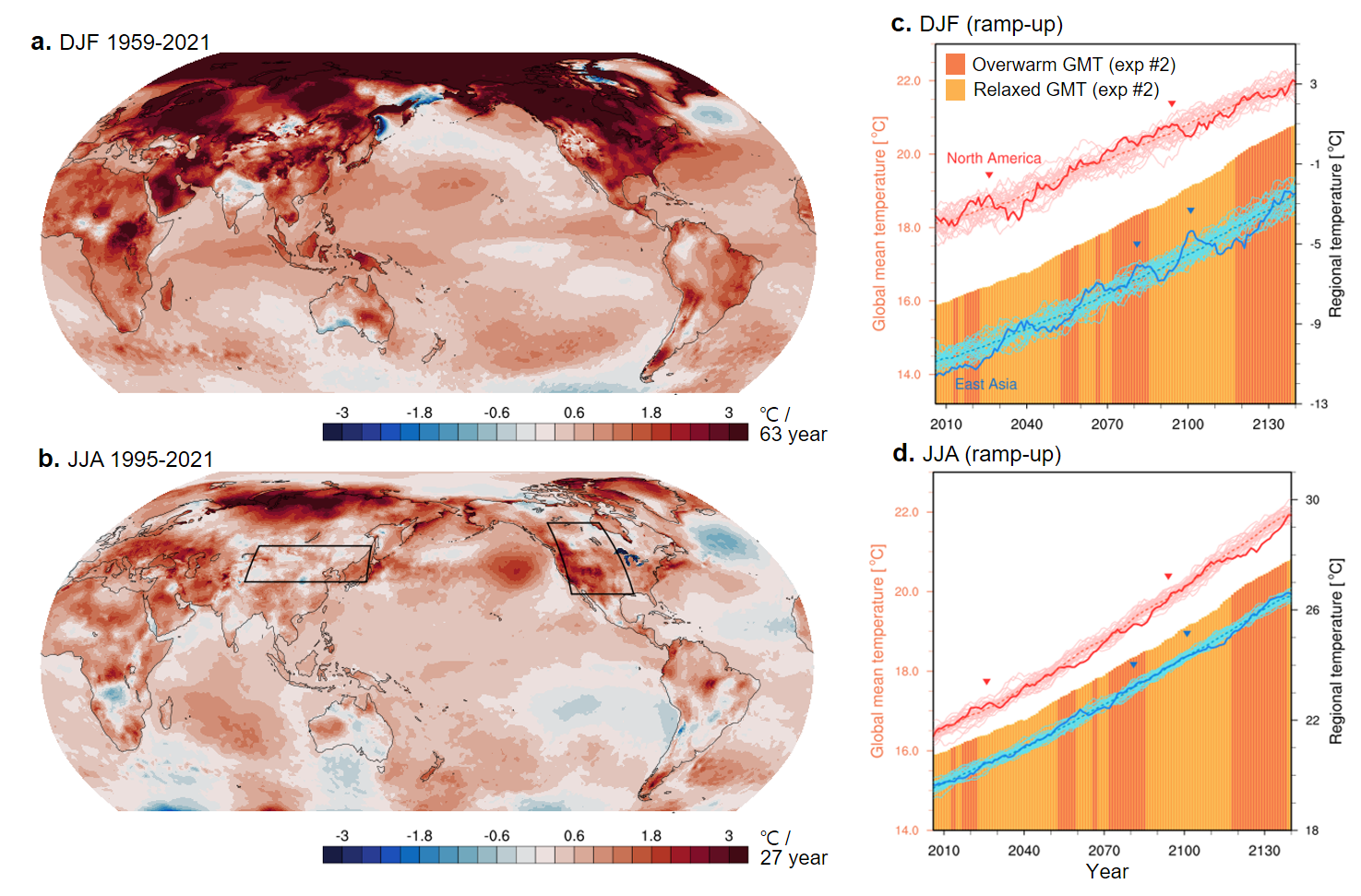


Supplementary Figure 1. Temperature trend in the observations and ramp-up experiments. a, b. Linear trend in observed temperatures for (a) the winter-mean (Dec-Feb) of 1959/60-2021/22 and (b) the summer-mean (Jun-Aug) of 1995-2021. c, d. An 11-year running-mean time series of (c) winter and (d) summer temperatures over North America (red) and East Asia (blue) in ramp-up experiments. Bright-colored lines represent each individual ensemble simulation in which the second ensemble member is highlighted in darker colors. Upside-down triangle denotes the onset of a local warming hiatus in a sample. Bars depict the annual-mean GMT (second ensemble member) highlighted by a darker color when warmer than the ensemble average.


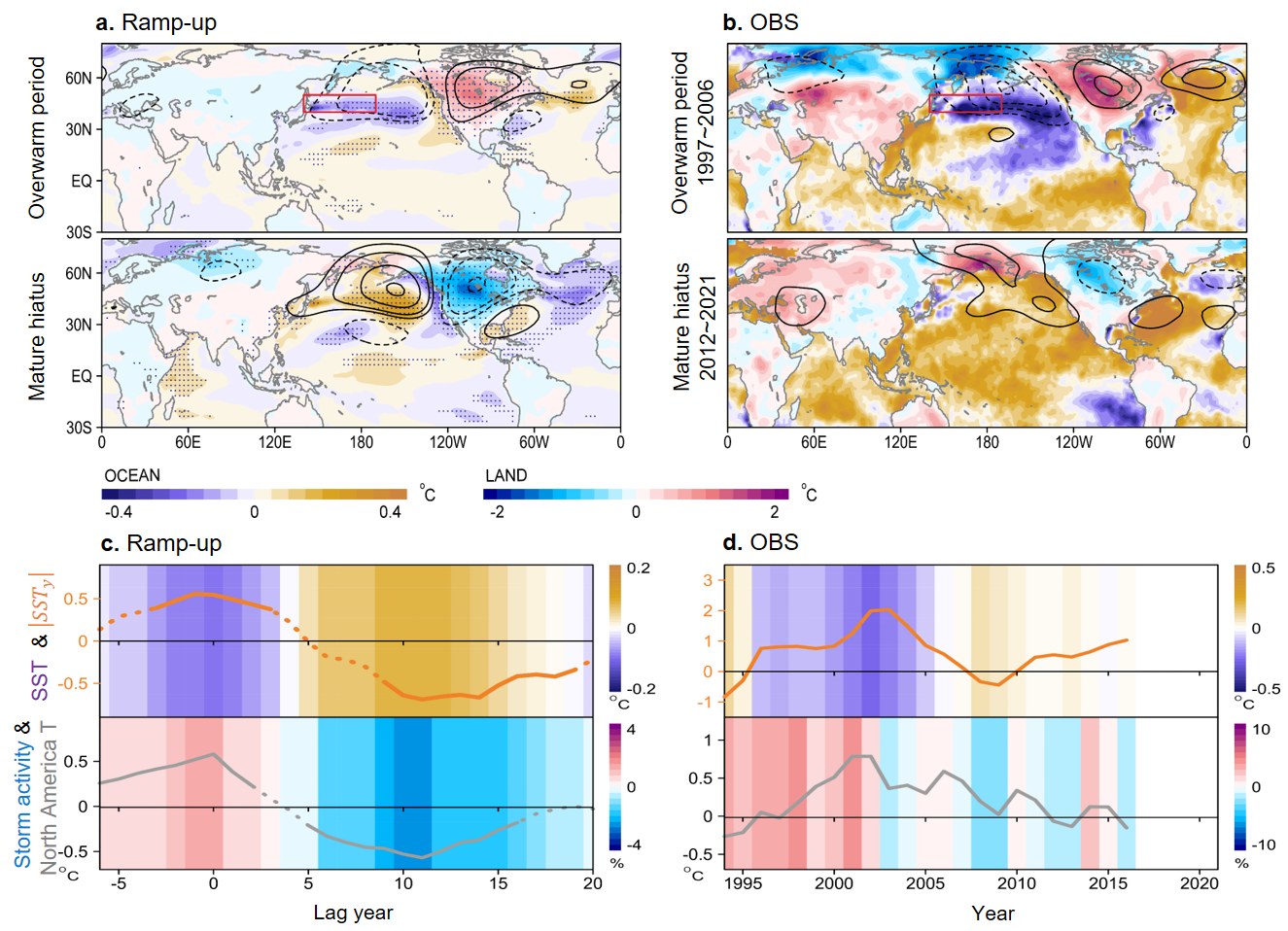


Supplementary Figure 2. Atmosphere and ocean conditions during the overwarm and mature hiatus decades of North America. a, b. Temperatures over land and ocean (shading; surface air temperature and SST) during the overwarm (upper) and mature hiatus (lower) periods in (a) ramp-up experiment and (b) observation. Contours denote the average atmospheric circulation in the upper-troposphere (300 hPa geopotential height anomaly) during each decade in which solid (dashed) lines represent anticyclonic (cyclonic) circulations (5 m interval for model output and 15 m interval for observation without zero line). c, d. (Upper) Time evolution of SST (11-year running average; shading) over the northern North Pacific (red boxed region in (a) and (b); 140°E -170°W, 40-50°N) and north-south SST gradient ($\left| \mathbf{SST}_{\mathbf{y}} \right|$) near the North Pacific gyre (brown curve; non-dimensionalized; see Methods and Supplementary Fig. 6). (Lower) Relative change of storm track activity anchored over the North Pacific gyre (shading; Methods) and North America temperature (grey curve). Stippling in (a) and the solid curve in (c) indicate values significant at the 95% confidence level. Data in the left column are derived from ramp-up experiments, and the right column presents the observational reference.


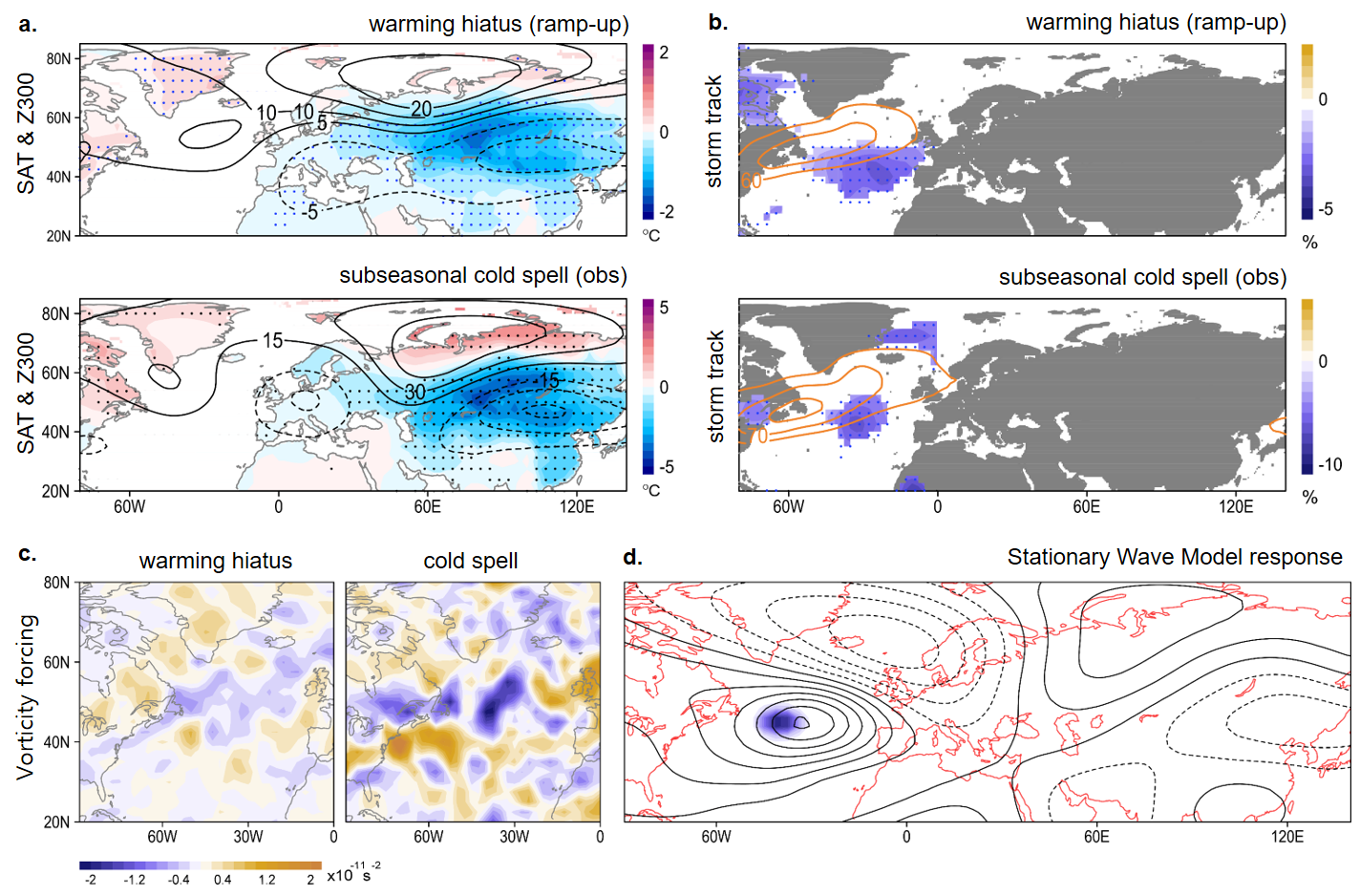


Supplementary Figure 3. Remote forcing for an East Asian cold period. a, b. Anomalous conditions related to an East Asian warming hiatus (upper; ramp-up) and winter cold spell (lower; observation) in terms of (a) geopotential height at 300 hPa (Z300; contour; gpm unit) and surface air temperature (SAT; shading) and (b) storm track change (shading; relative intensity (%) to climatology denoted by contour). Unlike the anomalies associated with warming hiatus obtained from ramp-up experiments, the anomalies associated with cold spell were computed via linear regression onto observational monthly temperatures over East Asia. c. Transient eddy vorticity forcing (see methods) associated with East Asian warming hiatus (left) and cold spell (right). d. Stationary wave response (contour; streamfunction) to point-wise divergent transient eddy vorticity forcing (shading) from the stationary wave model (Methods). Unlike the anomaly pattern shown in (a), anomalous background flow conditions over the North Atlantic driving the planetary wave response were not superimposed in this simple model output.


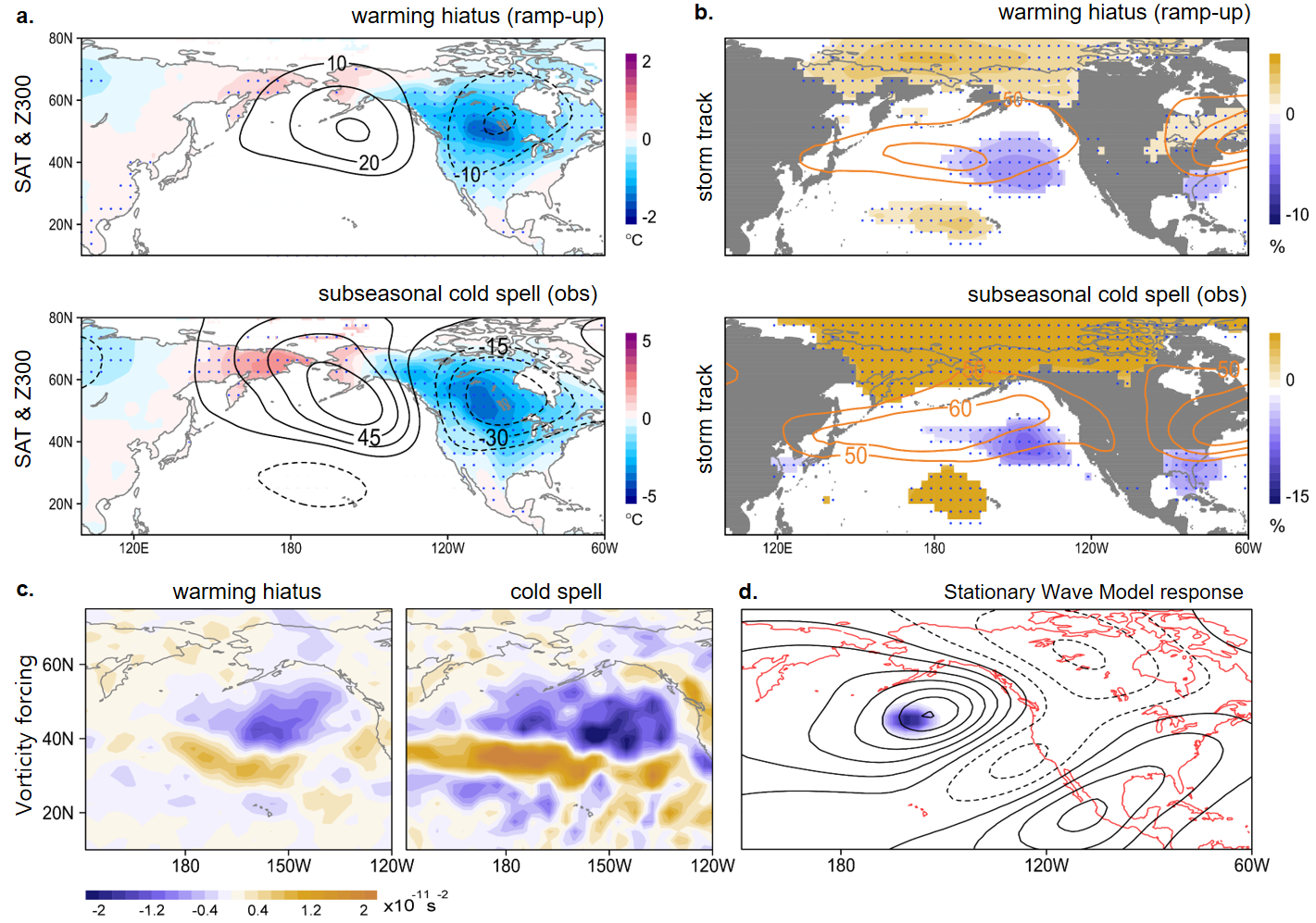


Supplementary Figure 4. Remote forcing for a North America cold period. a, b. Anomalous conditions related to a North American warming hiatus (upper; ramp-up) and winter cold spell (lower; observation) in terms of (a) geopotential height at 300 hPa (Z300; contour; gpm unit) and surface air temperature (SAT; shading) and (b) storm track change (shading; relative intensity (%) to climatology denoted by contour). Unlike the anomalies associated with warming hiatus obtained from ramp-up experiments, the anomalies associated with cold spell were computed via linear regression onto observational monthly temperatures over North America. c. Transient eddy vorticity forcing (see methods) associated with North America warming hiatus (left) and cold spell (right). d. Stationary wave response (contour; streamfunction) to point-wise divergent transient eddy vorticity forcing (shading) from the stationary wave model (Methods). Unlike the anomaly pattern shown in (a), anomalous background flow conditions over the North Pacific driving the planetary wave response were not superimposed in this simple model output.


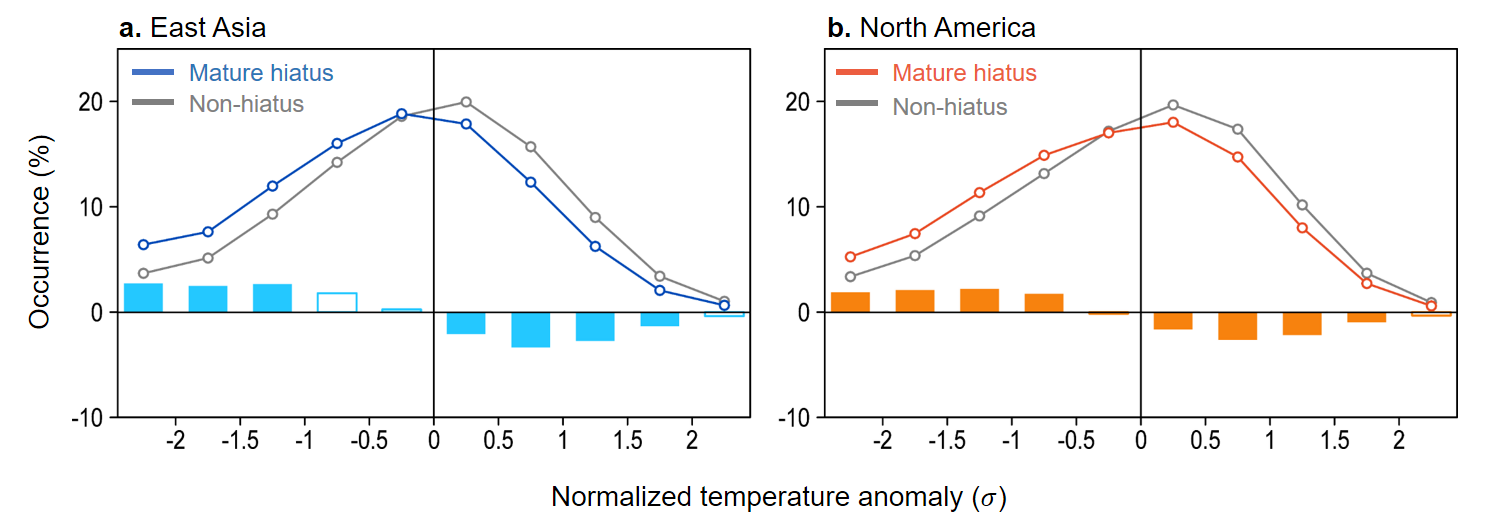


Supplementary Figure 5. Increased probability of cold extremes during a mature hiatus period. a, b. Daily surface air temperature distribution in (a) East Asia and (b) North America during a mature hiatus period (colored line) and non-hiatus period (gray line; Methods). Filled bars denote a significant difference between the two periods with a 95% confidence level tested by 5000 random resamples.


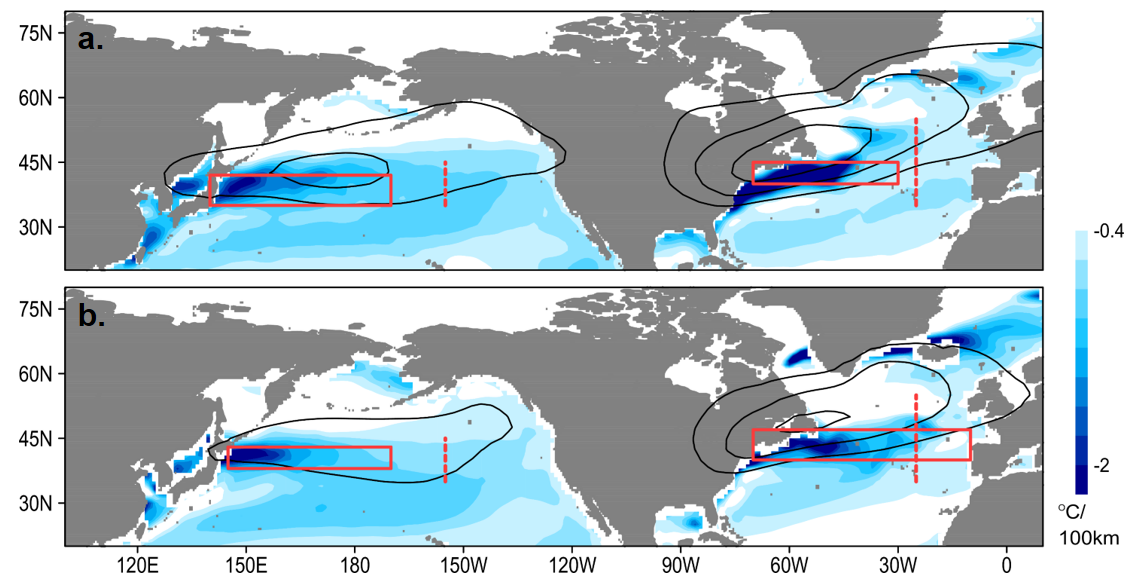


Supplementary Figure 6. SST gradient and storm track climatology. a, b. Winter climatological meridional SST gradient (shading;°C/100km) and climatological storm track intensity (contour; gpm) in the (a) observation and (b) ensemble mean winter climatology in ramp-up experiments (average for 2001-2020). The red box shows the concerned region in analyzing the SST gradient near the Kuroshio Extension (35-42°N, 140°E-190°W for observation and 38-43°N, 140°E-190°W for model) and Gulf Stream (40-45°N, 70-30°W for observation and 37-47°N, 70-10°W for model). Storm track activity was measured along the red dotted line (155°W, 35-45°N over the Pacific and 25°W, 35-55°N over the Atlantic).


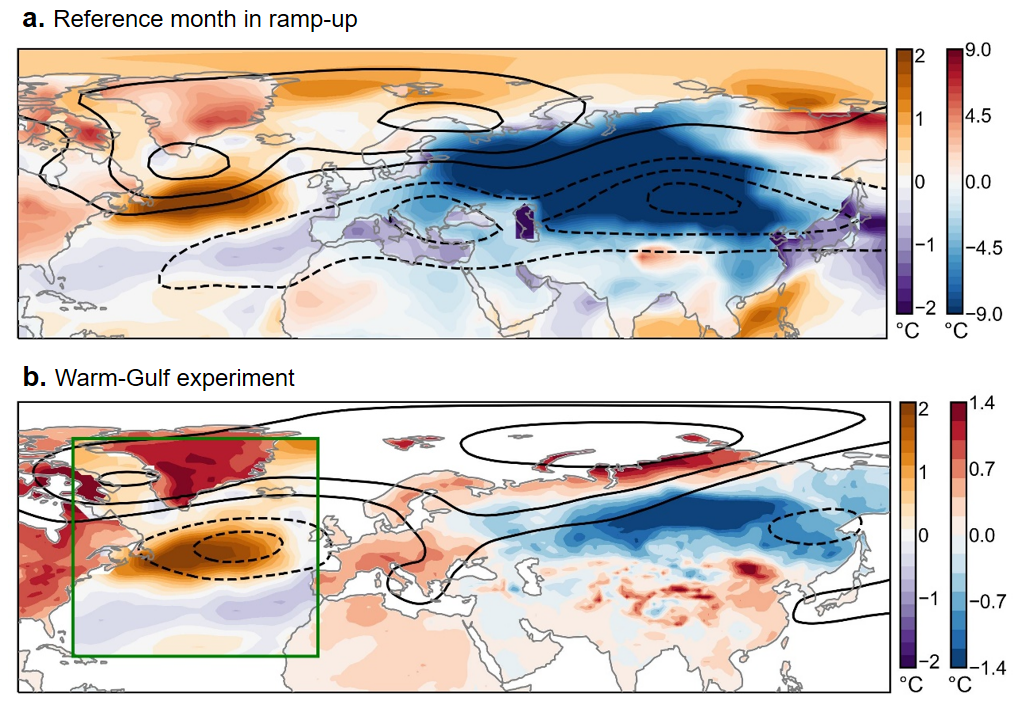


Supplementary Figure 7. Influences of the weakened North Atlantic frontal gradient. a. Temperatures over the ocean (purple-brown shading) and land (blue-red shading) during a reference period, chosen to force the Warm-Gulf experiment (Methods). Contours denote the corresponding atmospheric circulations (300 hPa geopotential height anomaly drawn with 80 gpm interval) b. Average response of planetary wave and temperature in the Warm-Gulf experiments (contours with 15 gpm interval). Values present deviations from the control experiment (Methods). The green box indicates the region where the anomalous SST forcing was imposed.
